# Blood pressure thresholds for the administration of balanced crystalloids and the effect on intra-operative hypotension: A proof-of-concept causal inference analysis featuring idealised dynamic treatment regimens

**DOI:** 10.1097/EJA.0000000000002356

**Published:** 2026-02-02

**Authors:** Markus Huber, Hyung-Chul Lee, Patrick Y. Wuethrich

**Affiliations:** From the Department of Anaesthesiology and Pain Medicine, Inselspital, Bern University Hospital, University of Bern, Bern, Switzerland (MH, PYW), Department of Anesthesiology and Pain Medicine, Seoul National University College of Medicine, Seoul National University Hospital, Seoul, South Korea (H-CL)

## Abstract

**BACKGROUND:**

Haemodynamic management and fluid administration constitute a challenging cornerstone of intra-operative care to counteract hypotension. Observational studies remain a vital source of evidence regarding the associations of fluids and vasopressors with peri-operative outcomes.

**OBJECTIVE:**

To evaluate dynamic treatment regimens (DTRs) of balanced crystalloids on the incidence of intra-operative hypotension. DTRs constitute a statistical framework to evaluate the causal impact of different treatment strategies (e.g. fluid administration) on an outcome (e.g. hypotension) whilst accounting for time-dependent treatments and treatment-confounder feedbacks.

**DESIGN:**

Analysis of observational data using modern causal inference methods.

**SETTING:**

Tertiary institution in South Korea during January 2011 to December 2020 (INSPIRE dataset).

**PATIENTS:**

*N* = 23 305 patients undergoing elective surgery under general anaesthesia.

**INTERVENTIONS:**

The DTRs refer to thresholds of mean invasive arterial blood pressure (MAP) to guide a hypothetical administration of 250 ml of balanced crystalloids. As example, a DTR implies administering 250 ml of balanced crystalloids if MAP drops below a 70 mmHg threshold, and to administer no crystalloids otherwise. MAP thresholds of 60, 65, 70 and 75 mmHg were evaluated.

**MAIN OUTCOME MEASURES:**

Primary endpoint was intra-operative hypotension defined as a MAP below 65 mmHg averaged in 15-min windows.

**RESULTS:**

The DTRs resulted in clinically similar incidences of intra-operative hypotension for surgeries shorter than 2 h. For surgeries lasting longer, the DTRs with early fluid administration resulted in a reduction of hypotension of more than 4% towards the end of the surgery. As higher MAP-thresholds imply higher amounts of fluid, our findings suggest there is in general a positive effect of fluid administration on intra-operative hypotension.

**CONCLUSIONS:**

Using modern causal inference methods, we demonstrated the clinical utility of idealised DTRs to study the impact of time-dependent haemodynamic management on the incidence of intra-operative hypotension in idealised settings. Future studies are required to investigate more complex DTRs mirroring clinical practice and to assess the robustness of the findings with respect to the different surgical populations.

**KEY POINTS**

- Haemodynamic management and fluid administration constitute a challenging cornerstone of intra-operative care to counteract hypotension.
- Using modern causal inference methods, we evaluated so-called dynamic treatment regimens (DTRs) of balanced crystalloids on the incidence of intra-operative hypotension using a large observational dataset in an idealised, proof-of-concept fashion.
- The methods take explicitly into account the time-varying treatment with balanced crystalloids and treatment-confounder feedbacks (e.g. with mean arterial pressure) underlying peri-operative care.
- For elective surgeries shorter than 2 h, the DTRs do not clinically differ in terms of hypotension, whereas for surgeries lasting longer, early fluid administration shows a benefit by possibly reducing the incidence more than 4%.
- As higher MAP-thresholds imply higher amounts of fluid, our findings suggest there is in general a positive effect of fluid administration on intra-operative hypotension.
- Sensitivity analyses highlighted that the impact of the DTRs on intra-operative hypotension differ with respect to the type of surgery and the type of crystalloid used, providing possible future research avenues to investigate surgery-specific DTRs with different types of fluids.

## Introduction

Haemodynamic management with fluid and vasopressor administration constitutes a cornerstone of intra-operative care.^1,2^ The major challenge is to avoid intra-operative hypotension (IOH) as these episodes are associated with severe adverse outcomes, including acute kidney injury, myocardial injury, stroke, delirium and mortality.^3^ Although several randomised controlled trials provide evidence regarding optimal blood pressure strategies, observational studies remain an essential source of evidence regarding the association of IOH and postoperative outcomes.^4^ In addition, they provide important evidence regarding the associations of certain types and amounts of fluids and vasopressors with postoperative outcomes.^5–7^

In observational studies, the analysis of haemodynamic management and its association with peri-operative outcomes is traditionally based on aggregated quantities, for example the duration under a certain mean arterial pressure (MAP) threshold, total fluid balance or cumulative norepinephrine dose.^7–9^ However, a key characteristic of time-dependent intra-operative care is that there are feedbacks between the treatments and time-varying confounders. For instance, fluids are administered depending on the patient's current haemodynamic state, for example judged with respect to the current MAP (among many other factors). A so-called treatment-confounder feedback is present when a quantity that guides treatment decisions (e.g. MAP) is influenced by the treatment (e.g. fluid administration) itself at a later stage.^10^ Importantly, these so-called treatment-confounder feedbacks require dedicated, statistical methods beyond traditional regression.^11^

In this context, there have been significant advances in the analysis of observational, longitudinal datasets featuring treatment-confounder feedbacks.^12^ These new statistical frameworks allow extending the analysis of single-point treatments to incorporate longitudinal treatments occurring at multiple time points, requiring the consideration of treatment *regimens* (or *policies* or *strategies*) instead of single treatments. Moving from a single point treatment towards treatment regimens where multiple treatments occur over a specified period – for example intra-operative – enables the investigation of clinically relevant interventions with respect to a time-varying variable which is relevant for treatment initiation, administration or continuation.^13^ These are referred to so-called dynamic treatment regimens (DTRs).^14,15^ For instance, a DTR could be to administer a certain drug at a particular time only if a critical variable drops below a certain threshold, for example if MAP drops below a threshold of 65 mmHg. Although causal inference methods to adjust for time-dependent confounding were employed in the domain of peri-operative care in the context of antihypertensive combination therapy, an analysis of DTRs for fluid administration is still lacking.^16^ In this context, a recent pilot study showed that patients assigned to personalised blood pressure management received more fluids than patients assigned to routine blood pressure management,^17^ thus motivating our investigation between MAP thresholds and fluid administration.

Therefore, the objective of this study is to investigate the causal impact of different DTRs on intra-operative hypotension using different MAP thresholds to administer an amount of balanced crystalloids. We further examine the implications of these dynamic treatment strategies in a clinical context with respect to the duration of surgery and compare the magnitude of the impact to a clinically relevant threshold.

## Materials and methods

### Data

We use data from the publicly available INSPIRE dataset (version 1.3).^18^ The dataset features peri-operative variables of patients undergoing surgery under general, neuraxial and regional anaesthesia, and monitored anaesthesia care between January 2011 and December 2020 at the Seoul National University Hospital, South Korea. The ethical statement related to the primary publication of the dataset is as follows: ‘This study was approved by the Institutional Review Board (IRB) of Seoul National University Hospital (SNUH) (IRB No. H-2210–078-1368). The IRB also waived the informed consent due to the retrospective nature of the study design. Additionally, the Institutional Data Review Board (DRB) of SNUH approved the release of the dataset to the public after confirming the adequacy of anonymisation (DRB No. BD-R-2022-11-02)’.^18^ The data are available on *PhysioNet* after completing a required training and signing a data use agreement.^19^

From the total of *N* = 130 960 interventions, we only included elective cases in general anaesthesia with invasive MAP measurements. We finally analysed 23 305 patients with available complete data for pre-operative variables (e.g. demographics, comorbidities, laboratory measurements) as well as for intra-operative variables (e.g. invasive MAP, heart rate, crystalloids, colloids and vasopressors). A detailed flowchart illustrating patient and variable selection including exclusion criteria is provided in Supplementary Figure SM1.

Intra-operative data was averaged in 15-min windows and we considered intra-operative data up to 4 h. The empirical choice of 15-min windows is based on methodological grounds. The causal inference methods used in this study require that there is a nonzero probability of receiving the intervention of interest – here the administration of balanced crystalloids – at each time point. This requirement is referred to as the positivity assumption.^12^

On the one hand, if we were to choose shorter time windows, for example 5-min windows, there could be instances where no patient received any balanced crystalloids. In such instances, we could make no inferences on what would have happened with respect to hypotension if some patients had received some crystalloids as no information regarding the treatment effect of crystalloids on hypotension is present during that time window. On the other hand, choosing longer time windows (e.g. 30 min) could feature multiple administrations of fluids and vasopressors, for example two boli of balanced crystalloids. The average effect of these two boli on the MAP could potentially mask the impact of each treatment on the incidence of hypotension. Thus, in long time windows, there is the risk that summary values of treatments and confounders (e.g. their mean values) do not properly account for the treatment-confounder feedbacks underlying the time-dependent treatment. We therefore chose 15-min windows to balance the different statistical requirements for the causal inference analysis for haemodynamic treatments, which is described in more detail below.

### Primary endpoint

The primary endpoint was IOH defined as an invasive MAP averaged in 15-min windows below 65 mmHg.

### Causal inference for longitudinal treatments

The objectives and methods of causal inference have already been described and summarised in the literature.^20–26^ Here, we focus on the analysis of a longitudinal, observational dataset featuring both time-independent and time-varying confounding variables as well as a time-dependent treatment. An essential characteristic of a causal inference approach is to draw the underlying cause-effect relationships between these random variables in a so-called directed acyclic graph (DAG) based on (untestable) domain knowledge.^12^

**Fig. 1:**
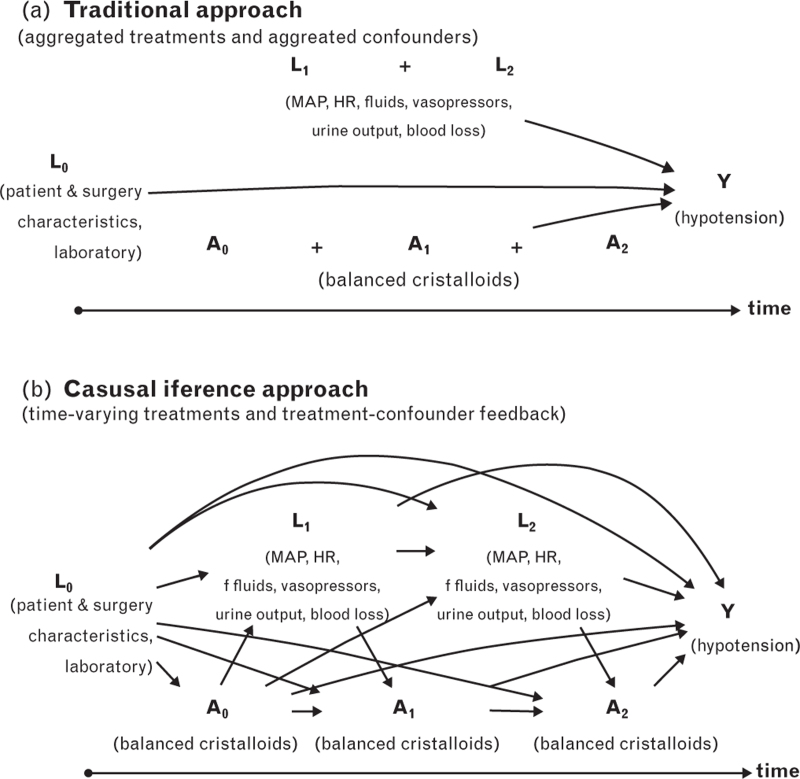
Illustration of the analysis methods for time-dependent haemodynamic management with balanced crystalloids administration as treatment variable (A) and hypotension as outcome (Y).

Figure 1 contrasts the assumed cause-effect relationships of a traditional approach with those of a causal inference approach when examining the effect of haemodynamic management on a clinical outcome. Figure 1a depicts the traditional approach where aggregated measures of both treatments (fluid administration) and confounders (e.g. MAP, vasopressors and urine output) are considered. There are no time-dependent feedbacks between treatment and confounders. Figure 1b depicts the time-dependent treatment with time-dependent confounders and treatment-confounder feedbacks. For example, baseline variables can influence the first fluid treatment (L_0_ → A_0_), which in turn may impact the first intra-operative assessment of haemodynamic stability (A_0_ → L_1_). Further fluid administration is both impacted from previous treatments and confounders (e.g. A_0_ → A_1_ and L_1_ → A_1_).

Baseline variables are collected pre-operatively (L_0_) and there are three intra-operative time points where crystalloids can be administered (A_0_, A_1_ and A_2_). Time-varying confounders potentially affecting the time-dependent treatments are collected at two time points (L_1_ and L_2_). (a) Traditional methods considering aggregated (e.g. averaged or cumulative) values of fluid administration and confounders. (b) Causal inference approach featuring treatment-confounder feedback.

### Dynamic treatment regimens

Dynamic treatment regimens refer to statistical frameworks to study and to compare the effects of different hypothetical interventions (or treatments) on an outcome of interest in a time-varying setting.^12,27^ They are hypothetical and counterfactual in the sense that they constitute valid and clinically relevant treatment regimens, but have not been explicitly assigned as treatments as in a randomised controlled trial (but potentially could be used as treatments in an actual trial). As such, observational datasets consisting of multiple and varying observed treatments – potentially including treatment regimens very similar or even identical to the regimen of interest – constitute the main source of information to model DTRs.

Overall, DTRs formalise the sequential decision-making of practitioners when administering time-dependent treatments: they model the statistical relationships over the course of multiple time points between variables that influence a practitioner's decision to choose a particular treatment, the intervention itself and the effect of previous and current treatment decisions on an outcome of interest. What sets DTR apart from static treatment regimens – which set the treatment options to a fixed level for all time points, for example to always treat with a particular intervention or to never treat – is that they explicitly incorporate the time-varying variables (confounders) for each patient and thus personalise the decision-making over the course of follow-up. Overall, DTRs were shown to provide clinically relevant information regarding treatment options in a variety of domains.^28^

More formally in the context of this study, DTRs denote hypothetical time-dependent interventions *d*_*t*_ that assign a treatment value according a patient's present or previous value of a covariate of interest (the so-called patient history).^29^ In our case, we consider the administration of 250 ml of balanced crystalloids only if the invasive MAP at a particular time point (*MAP*_*t*_) dropped below a predefined threshold *θ*:


$$d_{t}=\left\{\begin{array}{cc} 250\;mL & MAP_{t}<0\\ 0\;mL & otherwise \end{array}\right.$$

The 250 ml of balanced crystalloids is considered an idealised fluid administration, and we note that the exact amount of 250 ml was only administered a few times in the INSPIRE cohort investigated in this study. We consider clinically relevant MAP thresholds of *θ* = 60 mmHg, *θ* = 65 mmHg, *θ* = 70 mmHg and *θ* = 75 mmHg, encoding clinical approaches to fluid administration of treating early (*θ* = 75 mmHg) versus treating late (*θ* = 60 mmHg). Note that these thresholds were defined retrospectively. The balanced crystalloids are termed ‘plasma solution’ in the INSPIRE dataset and refer to the product Plasma Solution A (HK Inno N, Cheongju, South Korea). We chose the administration of balanced crystalloids as intervention, as these constituted the type of fluid which was administered most frequently (in 71% of the surgeries considered here; Table 1). We note that we did not include Hartmann's solution as balanced crystalloids to the ‘plasma solution’, as this would have resulted in combining two different fluid products. Throughout the manuscript, we refer to the product Plasma Solution A when using the term balanced crystalloids.

**Table 1:** Patients’ characteristics and summary measures of haemodynamic variables and haemodynamic management stratified according to the administration of balanced crystalloids at some point during surgery

	All	No balanced crystalloids	With balanced crystalloids	*P*
				
	*N* = 23 305 (100%)	*N* = 6775 (29%)	*N* = 16 530 (71%)	
Demographics				
Age (years)	60.0 [45.0;70.0]	60.0 [50.0;70.0]	60.0 [45.0;70.0]	<0.001
Sex (female)	12 233 (52.5%)	3589 (53.0%)	8644 (52.3%)	0.352
BMI (kg/m^2^)	23.4 [21.5;26.0]	23.4 [20.8;25.7]	23.8 [21.5;26.0]	<0.001
American Society of Anesthesiologists physical status				
1	6745 (28.9%)	2096 (30.9%)	4649 (28.1%)	
2	13 541 (58.1%)	3729 (55.0%)	9812 (59.4%)	
3	2881 (12.4%)	914 (13.5%)	1967 (11.9%)	
4	133 (0.6%)	35 (0.5%)	98 (0.6%)	
5	5 (<0.1%)	1 (<0.1%)	4 (<0.1%)	
Surgery characteristics				
Department				<0.001
General surgery	6’293 (27.0%)	2990 (44.1%)	3303 (20.0%)	
Other	17 012 (73.0%)	3785 (55.9%)	13 227 (80.0%)	
Surgery duration (min)	160.0 [105.0;250.0]	135.0 [75.0;230.0]	170.0 [115.0;255.0]	<0.001
Anaesthesia duration (min)	210.0 [145.0;300.0]	175.0 [115.0;275.0]	220.0 [155.0;310.0]	<0.001
Hospitalisation (days)	8.0 [6.0;12.0]	9.0 [5.0;12.5]	8.0 [6.0;12.0]	0.008
Intra-operative averaged haemodynamics				
Arterial mean blood pressure (mmHg)	80.9 [74.2;87.3]	80.9 [73.2;87.6]	80.9 [74.4;87.2]	0.055
Heart rate (/min)	66.8 [59.3;75.0]	66.4 [58.7;74.9]	67.0 [59.5;75.0]	0.001
Pre-operative laboratory measurements				
Albumin (g/dl)	4.2 [4.0;4.4]	4.2 [4.0;4.4]	4.2 [4.0;4.4]	<0.001
Alkaline phosphatase (IU/l)	64.0 [52.0;80.0]	64.0 [52.0;80.0]	64.0 [52.0;80.0]	0.134
Alanine transaminase (IU/l)	18.0 [13.0;27.0]	17.5 [13.0;27.0]	18.0 [13.0;27.0]	<0.001
Activated partial thromboplastin time (sec)	31.2 [29.1;33.4]	31.2 [29.1;33.8]	31.2 [29.1;33.2]	0.081
Aspartate transaminase (IU/l)	21.0 [17.0;26.0]	21.0 [17.0;26.0]	21.0 [17.0;26.0]	<0.001
Blood urea nitrogen (mg/dl)	13.0 [11.0;17.0]	13.0 [11.0;17.0]	13.0 [11.0;17.0]	0.793
Calcium (mg/dl)	9.1 [8.8;9.4]	9.1 [8.8;9.4]	9.1 [8.9;9.4]	0.013
Chloride (mmol/l)	103.0 [102.0;106.0]	103.5 [102.0;106.0]	103.0 [101.5;106.0]	<0.001
Creatinine (mg/dl)	0.8 [0.7;0.9]	0.8 [0.7;0.9]	0.8 [0.7;0.9]	<0.001
Glucose (mg/dl)	105.0 [94.0;120.0]	101.0 [91.0;115.0]	105.0 [96.0;120.0]	<0.001
Haemoglobin (g/dl)	13.2 [12.2;14.2]	13.2 [11.9;14.2]	13.2 [12.2;14.2]	<0.001
Haematocrit (%)	38.6 [35.2;42.0]	38.5 [35.2;42.0]	38.6 [35.4;42.0]	0.539
Phosphorus (mg/dl)	3.5 [3.2;3.9]	3.5 [3.2;3.9]	3.5 [3.2;3.9]	0.281
Platelet count (/nl)	233.0 [194.5;274.5]	233.0 [189.0;273.0]	233.5 [200.0;282.0]	<0.001
Potassium (mmol/l)	4.0 [3.8;4.2]	4.0 [3.8;4.2]	4.0 [3.8;4.2]	<0.001
Prothrombin time (INR)	1.0 [0.9;1.0]	1.0 [0.9;1.0]	1.0 [0.9;1.0]	<0.001
Sodium (mmol/l)	139.0 [137.5;140.0]	139.0 [137.0;140.0]	139.0 [137.5;140.0]	0.812
Total bilirubin (mg/dl)	0.6 [0.5;0.8]	0.7 [0.5;0.8]	0.6 [0.5;0.8]	<0.001
Total protein (g/dl)	7.2 [6.8;7.5]	7.1 [6.8;7.4]	7.2 [6.8;7.5]	<0.001
White blood cell count (/nl)	6.3 [5.2;7.5]	6.1 [5.2;7.5]	6.3 [5.2;7.5]	<0.001
Fluids and vasopressors				
Half normal saline (Yes)	77 (0.3%)	73 (1.1%)	4 (<0.1%)	<0.001
If administered, amount (ml)	950 [550;1150]	950 [550;1150]	675 [525;925]	0.498
Hartmann's solution (Yes)	12 670 (54.4%)	3994 (59.0%)	8676 (52.5%)	<0.001
If administered, amount (ml)	450 [300;700]	600 [400;850]	400 [250;600]	<0.001
Normal saline (Yes)	6682 (28.7%)	1785 (26.3%)	4897 (29.6%)	<0.001
If administered, amount (ml)	400 [250;650]	450 [250;800]	400 [250;600]	<0.001
Balanced crystalloids (Yes)	16 530 (70.9%)	0 (0%)	16’530 (100%)	<0.001
If administered, amount (ml)	600 [300;1000]	600 [300;1000]	.	.
Hydroxyethyl-starch solution (Yes)	3712 (15.9%)	1196 (17.7%)	2516 (15.2%)	<0.001
If administered, amount (ml)	500 [400;600]	500 [400;800]	500 [500;600]	0.953
Total crystalloids and colloids (Yes)	21 498 (92.2%)	4968 (73.3%)	16 530 (100%)	<0.001
If administered, amount (ml)	1000 [550;1’550]	710 [400;1250]	1050 [600.0;1600]	<0.001
Epinephrine (Yes)	116 (0.5%)	11 (0.2%)	105 (0.6%)	<0.001
If administered, amount (ml)	20 [10;50]	20 [10;20]	20 [20;55]	0.051
Phenylephrine (Yes)	4166 (17.9%)	483 (7.1%)	3683 (22.3%)	<0.001
If administered, amount (ml)	70 [30;140]	60 [30;120]	70 [30;150]	0.002
Vasopressin (Yes)	103 (0.4%)	13 (0.2%)	90 (0.5%)	<0.001
If administered, amount (ml)	0.3 [0.1;0.6]	0.1 [0.1;0.5]	0.3 [0.2;0.7]	0.044
Ephedrine (Yes)	6878 (29.5%)	1419 (20.9%)	5459 (33.0%)	<0.001
If administered, amount (ml)	10 [5;15]	10 [5;15]	10 [5;15]	0.354
Transfused fresh frozen plasma (Yes)	214 (0.9%)	56 (0.8%)	158 (1.0%)	0.388
Transfused red blood cell (Yes)	474 (2.0%)	131 (1.9%)	343 (2.1%)	0.520
Fluid output				
Urine output (ml)	140.0 [20.0;330.0]	80.0 [0.0;240.0]	155.0 [50.0;375.0]	<0.001
Estimated blood loss (ml)	140.0 [40.0;330.0]	40.0 [0.0;200.0]	180.0 [60.0;390.0]	<0.001

Note that in this study, we restrict the analysis to surgery times up to 4 h. Categorical variables are summarised with counts and frequencies and numerical variables with medians and interquartile ranges.

Drawing valid inferences about the DTRs from an observational dataset requires the assumptions of positivity and weak sequential randomisation, which we assume to hold here.^14,15,30^ Briefly, the assumptions imply that there is no unmeasured confounding at each time point and that there is a nonzero probability of receiving fluid treatment at each of the 15-min windows, which is valid for our choice of 15-min windows (Fig. 3). Supplementary Figure SM2 provides a detailed step-by-step explanation of the steps involved in a simplified DTR of haemodynamic management and its effect on hypotension. The simplified DTR illustrates that modern causal inference methods may employ the same statistical methods as traditional analyses (e.g. multivariable regression) but do so to adjust for confounding at each time step during follow-up and to compute counterfactual outcomes.

### Estimation

The causal impact of the DTRs on intra-operative hypotension is computed with a targeted maximum likelihood estimator (TMLE), using a random forest for the outcome regression and for the estimation of the exposure mechanism.^31–33^ We adjusted for baseline confounders (age, sex, BMI and American Society of Anesthesiologists physical status, surgery department and pre-operative laboratory measurements; Table 1) and time-varying confounders averaged in 15-min windows (MAP, heart rate, colloids, crystalloids, vasopressors, transfused red blood cells and fresh frozen plasma, urine output and estimated blood loss; Table 1). Crystalloids include half normal saline (0.45% sodium chloride), normal saline (0.9% sodium chloride), Hartmann's solution and colloids include hydroxyethyl-starch solution. Vasopressors include epinephrine, phenylephrine, vasopressin and ephedrine. The starting point of the DTR (‘time zero’) was chosen as the start of surgery. Drop-outs related to different surgery durations were not considered as informative censoring.

We computed the MAP-based dynamical treatment regimens for each of the 15-min windows, resulting in a total of 5 (observed treatments plus 4 DTRs) x 16 (15-min windows for surgeries lasting up to 4 h) = 80 effect estimations. At each time window, say 120 min, we considered all patients whose surgery lasted at least that amount of time. For each time window, the associated estimate of the incidence of hypotension was shown with mean and 95% confidence intervals (CIs) for each DTR. We evaluated the clinical implications of the DTRs on the incidence of intra-operative hypotension by means of an empirically chosen clinically relevant difference of ±4%. That is, if a dynamic treatment results in a change in the observed incidence of hypotension at a particular intra-operative time point beyond ±4%, we consider the DTR to have clinically relevant effect on hypotension.

### Sensitivity analyses

We performed several sensitivity analyses to investigate both robustness and range of results depending on the type of surgery and the type of crystalloid used as hypothetical intervention. In the first case, we repeated the entire analysis, selecting patients only from the following surgeries: general, cardiothoracic, neurosurgery and urology. In the second sensitivity analysis, we chose Hartmann's solution as intervention in the dynamical treatment regime using the entire cohort.

### Summary measures, statistical testing and software

Categorical variables were summarised with counts and frequencies. Numerical variables were summarised with medians and interquartile ranges (IQRs). Baseline and intra-operative characteristics were stratified according to the administration of balanced crystalloids and compared by means of a chi-square test or Wilcoxon rank sum test, depending on a variable's distribution. No formal hypothesis testing based on a significance threshold, for example, 0.05, was performed.

### Software

All computations were performed with R version 4.2.3.^34^ The longitudinal treatment regimens were computed with the *lmtp* package.^31^

## Results

Table 1 summarises the demographics, comorbidities and haemodynamic summary measures of the 23 305 patients included in this study. Median age was 60 years and 52.5% were female. The majority of patients had ASA status II (58.1%). Median duration of surgery and anaesthesia was 160 min (IQR: 105 to 250 min) and 210 min (IQR: 145 to 300 min), respectively. Median MAP and heart rate was 81 mmHg (IQR: 74 to 87 mmHg) and 67 bpm (IQR: 59 to 75 bpm). Plasma solution was the most frequently administered intra-operative fluid in 71% of the patients considered here, followed by Hartmann's solution (54%) and normal saline (29%). Median administration of total crystalloids and colloids in patients received either one of them was 1000 ml (IQR: 550 to 1500 ml). With respect to vasopressors, ephedrine was most frequently administered in 30% of surgeries, followed by phenylephrine (18%). Patients who received balanced crystalloids at some point during the surgery were older, had longer surgeries and received overall more crystalloids and colloids (median of 1050 versus 710 ml). No albumin or dextrose was administered. The majority of surgeries were from general surgery (27%), cardiothoracic surgery (20%), and neurosurgery (19%) as shown in Supplementary Table SM3.

**Fig. 2:**
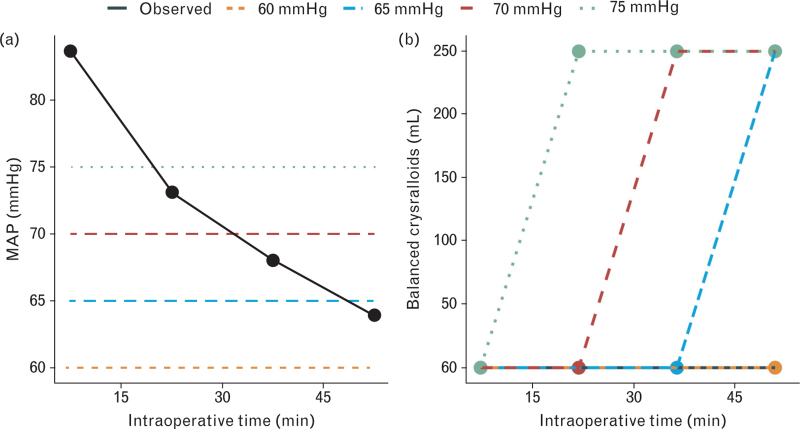
Illustration of the dynamic treatment regimens in one patient.

Figure 2 illustrates the set of dynamic crystalloids regimens for a selected patient undergoing surgery for 60 min. For simplicity, a patient with a short surgery duration was chosen. From its initial value of 84 mmHg, the MAP decreased over time to 64 mmHg, thus crossing the predefined MAP thresholds of 75, 70 and 65 mmHg. In the original dataset, the patient did not receive balanced crystalloids (Fig. 2b). However, under a DTR with a MAP threshold 75 mmHg, the clinician would have administered 250 ml of crystalloids at the second time step (between 15 and 30 min). For a MAP threshold of 70 mmHg, no crystalloids would have been administered for the first two time steps, but between 30 and 45 min after the start of surgery.

(a) Observed invasive mean arterial blood pressure (MAP) and MAP-thresholds of the four DTRs considered in this study. (b) Balanced crystalloids administration as a function of intra-operative time-dependent, observed MAP values. These values correspond to the fluid amount that a clinician would have administered in a hypothetical dynamical treatment regime.

**Fig. 3:**
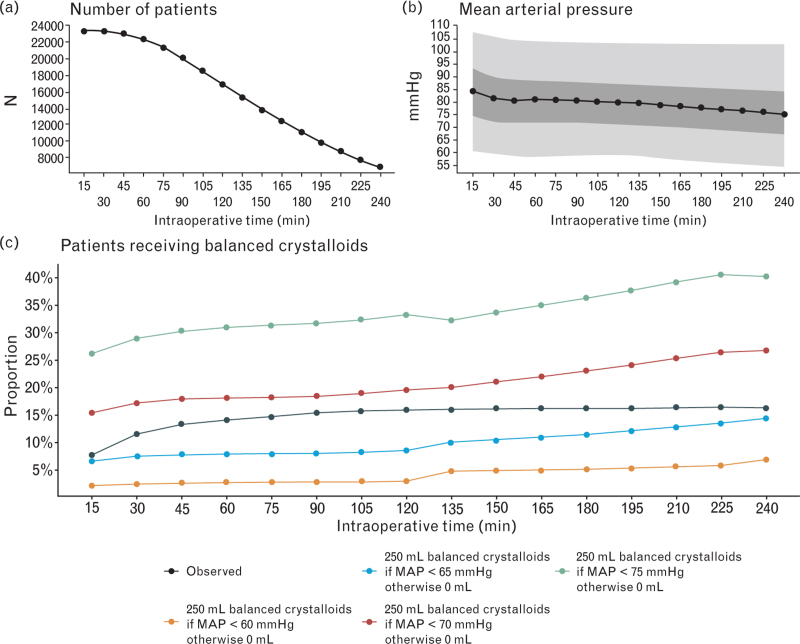
Cohort overview.

(a) Number of patients available for analysis at each time point. (b) Invasive mean arterial blood pressure (median, interquartile range und central 95% interval). (c) Proportion of patients receiving balanced crystalloids both in the observational dataset (black) and under the four dynamic treatment regimens considered in this study.

A cohort overview with key characteristics is shown in Figure 3. The number of patients available for analysis depends on surgery duration: Although more than 20 000 patients could be analysed for intra-operative times ranging between 1 and 1.5 h, less than 10 000 patients had surgery durations between 3 and 4 h (Fig. 3a). The median MAP decreased from around 85 mmHg to about 75 mmHg for surgeries lasting up to 4 h (Fig. 3b). Overall, the distribution of MAP in patients was similar in patients receiving balanced crystalloids and in those who did not receive balanced crystalloids (Supplementary Figure SM3). The proportion of patients who received balanced crystalloids both observed and under the different dynamical treatment regimens is illustrated in Fig. 3c: Between 5% and 15% received balanced crystalloids at some point in the observational dataset. When considering the hypothetical treatment regimens, Fig. 3c further highlights that the higher the MAP threshold is chosen – that is, the earlier crystalloids are administered – the higher the proportion of patients receiving crystalloids. Note that the proportion of patients with crystalloids administration increases for longer surgery durations as the invasive MAP decreases over time according to Fig. 3b.

**Fig. 4:**
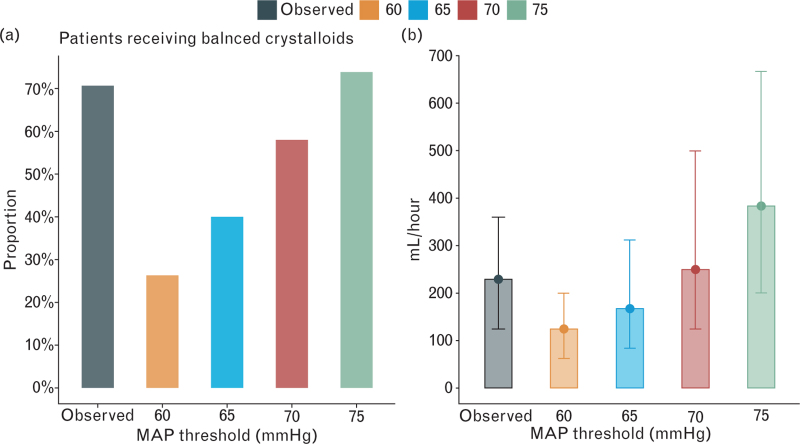
Implications of the dynamic treatment regimens (DTRs) on (a) Percentage of patients receiving balanced crystalloids both observed and under the DTRs and (b) Observed balanced crystalloids administration and fluid administration implied by the DTRs per hour.

The overall implications of the DTRs over the course of surgery for balanced crystalloids administration are shown in Fig. 4. Under a DTR with a threshold of 60 mmHg, only 27% of patients received balanced crystalloids at some point whereas 74% would have been treated with balanced crystalloids for the DTR with a 75 mmHg MAP-threshold (Fig. 4a). The DTR with a 75 mmHg MAP-threshold corresponded closely to the observed number of treated patients in our study sample. The median amount of balanced crystalloids varied considerably from a median administration of 125 ml/h (60 mmHg MAP-threshold) to 384 ml/h (75 mmHg MAP-threshold). The median amount administered under the dynamic treatment regime with a 70 mmHg MAP-threshold is close to the observed amount of 229 ml/h (Fig. 4b).

Medians and interquartile ranges are shown.

**Fig. 5:**
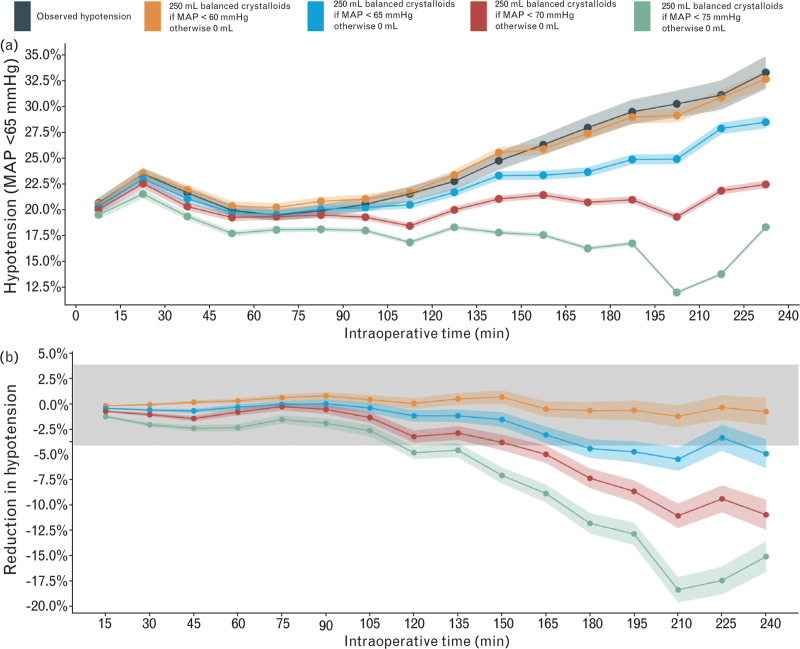
Observed incidence of intra-operative hypotension and implications of the balanced crystalloids treatment regimens on hypotension.

Figure 5 illustrates the impact of the DTRs on intra-operative hypotension. Note that each estimate refers to all patients with surgeries lasting at least as long as a particular time point. For example, the estimate of the observed hypotension incidence at 120 min includes all patients with surgeries lasting up to 2 h or longer. The observed incidence of hypotension ranges from 20.7% (95% CI: 20.2 to 21.2) during the first minutes of surgery up to 33.4% (95% CI: 31.8 to 35.0) for surgeries lasting 4 h (Fig. 5a). A detailed summary table with hypotension incidences for each time point and associated fluid administrations and measurements (e.g. balanced crystalloids, urine output) are provided in Supplementary Table SM4.

Means and 95% confidence intervals are shown. (a) Incidence of hypotension as defined as an invasive mean arterial pressure below 65 mmHg averaged over 15-min windows. (b) Reduction in hypotension implied by the dynamic treatment regimens with respect to the observed incidence. The grey shaded band denotes the clinically relevant difference of ± 4%.

The results of the sensitivity analyses are reported in Supplementary Figure SM5. The impact of the DTRs on intra-operative hypotension are similar for interventions in general surgery, cardiothoracic surgery and urologic surgery to the overall case described above in the sense that providing balanced crystalloids early (i.e. for higher MAP thresholds) results in a reduction of intra-operative episodes. In contrast, the DTRs for interventions in neurosurgery indicate much less sensitivity to the administration of balanced crystalloids. Note, however, that the number of patients as well the number of surgeries with longer duration, for example 2 h, is reduced in these sensitivity analyses. In additional, when using Hartmann's solution as the hypothetical intervention, intra-operative hypotensive events are reduced to a stronger degree as compared with balanced crystalloids (plasma solution) as shown in Supplementary Figure SM5.

We found that the earlier balanced crystalloids are administered – that is, a high MAP-threshold is chosen in the DTR – the less intra-operative hypotension occurs. As higher MAP-thresholds imply higher amounts of fluid (Figs. 3,4), Fig. 5a highlights that there is in general a positive (causal) effect of fluid administration on IOH. In addition, we found a strong time-dependent effect: for surgeries shorter than 2 h, the DTRs do not clinically differ, whereas for surgeries lasting longer, early fluid administration provides a benefit (Fig. 5b). Overall, the treatment regime of administering 250 ml only when intra-operative MAP drops below 60 mmHg results in a similar incidence of hypotension as observed in the dataset.

## Discussion

Intra-operative haemodynamic management frequently features time-dependent multiple treatment decisions based on time-varying confounding variables. This so-called treatment-confounder feedback poses challenges for the analysis of treatment effects with traditional statistical methods.^12^ DTRs are a promising application of a variety of statistical methods more suitable for such analyses and could play a role to inform clinically relevant decisions and optimal treatment strategies.^14,35^ Here, we applied for the first time DTRs to retrospectively evaluate implications of clinically motivated, hypothetical fluid administration regimens on IOH in a large, public observational dataset.

A key feature of the DTRs is that they explicitly account for the possible treatment-confounder feedback of multiple variables with the time-dependent administration of balanced crystalloids, including other crystalloids, colloids, vasopressors, blood products (red blood cells and fresh frozen plasma) as well as urine output and estimated blood loss. The DTRs further aim to mimic clinically relevant fluid therapies: The amount of 250 ml balanced crystalloids can be considered a typical fluid quantity; for example, fluid challenges often consisted of colloid boli of 250 or 500 ml administered in about 10 min, which are of similar magnitude both with respect to volume and time as in this study.^36^ Furthermore, a MAP threshold between 60 and 75 mmHg spans the threshold range employed in randomised controlled trials, when comparing hypotension avoidance treatment regimens (MAP target ≥ 80 mmHg) and hypertension-avoidance strategies (MAP target ≥60 mmHg).^37^ The choice of MAP thresholds further mirrors recent recommendations to maintain intra-operative MAP at least 60 mm Hg in at-risk patients.^38^ As the MAP was similarly distributed in patients receiving balanced crystalloids and in those not receiving balanced crystalloids (Supplementary Figure SM3), we emphasise that other quantities apart from the intra-operative MAP were the likely drivers for decisions regarding fluid administration. Therefore, the DTRs considered are idealised haemodynamic treatment regimens, and we consider the study as a proof-of-concept application of modern causal inference methods to account for time-dependent treatments with time-varying confounders.

The main result of this study is the demonstration of the causal effect of reducing the incidence of IOH by increased intra-operative administration of balanced crystalloids using an observational dataset. The DTRs allowed us to further investigate this causal effect over the course of the surgery (Fig. 5), resulting in clinically relevant insights. We found that for intra-operative times under around 2 h, different MAP thresholds result in clinically similar incidences of hypotension. In addition, the sensitivity analyses highlighted that the impact of the DTRs on intra-operative hypotension differ with respect to the type of surgery and type of crystalloids used. The sensitivity to the type of surgery mirrors clinical practice, as different interventions require tailored fluid approached, for example with respect to anticipated blood loss and type of crystalloid. Overall, these sensitivity results provide possible future research avenues to investigate surgery-specific DTRs with different types of fluids.

Fluid administration plays a central role in the management of IOH, as hypovolemia is a common and reversible cause of IOH. Adequate intravascular volume is essential to maintain cardiac preload, stroke volume and tissue perfusion, making fluid resuscitation a logical first-line measure when IOH occurs. However, not all IOH is due to volume depletion; factors such as anaesthetic-induced vasodilation, haemorrhage or impaired myocardial function may also contribute. Over longer surgeries, the role of fluid administration becomes more nuanced. Early in a procedure, a fluid bolus may quickly correct IOH from anaesthetic induction or minor blood loss. As operative time extends, cumulative factors (such as ongoing bleeding, redistribution of fluids from intravascular to extravascular and insensible losses) must be weighed against the risks of fluid overload, including tissue oedema, pulmonary compromise and delayed recovery. Thus, while fluid administration remains important throughout, its relevance shifts from being a simple first-line intervention to part of a more balanced strategy, often supplemented by vasopressors, blood products and advanced haemodynamic monitoring to maintain stability with precision. As the definition and the choice of treatment of IOH is still debatable, it is still unclear which interventions clinically improve outcomes. Here, the longer the surgery, the more the choice of MAP-threshold for fluid administration mattered with respect to hypotension (Fig. 5). This is of relevance, as, for example, acute kidney injury and cardiac events are major postoperative outcomes associated with hypotension in noncardiac surgery patients.^1^ This observation can also be explained by the pharmacokinetics of the fluids administered *per se*, as the blood volume expansion of the crystalloids is poor (around 20%).^39^

Overall, causal inference based methods such as DTR play an important role in multiple domains, for example in Epidemiology, and provide clinically relevant insights regarding treatment options: for instance when to initiate treatment of combined antiretroviral therapy in HIV-infected patients.^14,27^ Given the causal structure of peri-operative care with time-dependent treatments and with treatment-confounder feedbacks (Fig. 1), the domain of Anaesthesiology is likely to benefit from these modern statistical methods as well. For example, DTRs allow to evaluate a larger number of possible interventions than are likely feasible in a multiarm randomised trial.^12^ As such, they provide an invaluable tool for hypothesis testing of diverse haemodynamic strategies in many different populations and surgical settings. The findings of these studies could inform future randomised controlled trials with respect to efficacy and feasibility of different haemodynamic treatment regimens. Further applications of framework applied here could feature the analysis of patient-specific blood pressure targets or threshold with respect to time-weighted averages of hypotension.^8,40,41^

This study features inherent limitations. Chiefly amongst those are the facts that we had to restrict the computations as well as the identification of the causal effects to the set of variables collected in the INSPIRE dataset and the haemodynamic management is represented in an idealised fashion. For instance, we note that the decision to administer fluids is not only based on MAP but on a variety of other clinical indicators such as pulse pressure variation and arterial lactate. We emphasise that the DTRs based on MAP only constitute simplified interventions. However, we would argue that they provided clinically relevant insights into the possible causal effect of fluid administration strategies on time-dependent IOH. Assuming that the variables collected in the INSPIRE dataset fulfil the assumptions required to identify the causal estimates of this study (incidence of hypotension), one can consider the effect of different DTRs on hypotension as ‘causal’. However, future sensitivity analyses could analyse the robustness with respect to unmeasured confounding in more detail. In addition, the data collection has further implications of the analysis, for example if and how often certain time-varying confounders such as fluids or vasopressors were collected. Therefore, there are additional risks for unmeasured confounding and for selection bias. Further, the population underlying the INSPIRE dataset as well as the clinical practices related to haemodynamic management in this tertiary institution makes comparisons to other institutions challenging. Another limitation is the choice of 15-min window for the analysis. Although clinically motivated, irregular assessment times would require additional methods.^42^ A further important caveat is that the current set of DTRs does not incorporate possible adverse effects of high fluid administrations, for instance in the case of a DTR based on a MAP-threshold of 75 mmHg.^43^ In addition, the analysis framework here does not account for different causes of IOH with regard to the phase of surgery: We considered a single causal effect of balanced crystalloids on hypotension and could not differentiate the mechanisms involved in postinduction hypotension and early-IOH.^44^ We further note that our selection of inclusion criteria (Supplementary Figure SM1) could result in selection bias.

In conclusion, we adopted a causal inference framework for the first time to investigate the causal effect of different fluid administration regimens based on target MAP on IOH using observational data. We demonstrated a reduction in time-dependent IOH for higher balanced crystalloids regimens. Further studies are required to investigate the effect of these DTRs on postoperative outcomes, for example acute kidney injury, myocardial injury or stroke. In addition, method comparison studies between traditional analyses based on aggregated quantities and causal inference methods featuring time-dependent treatment-confounder feedback may lead to clinically relevant insights into the relative effect sizes of different haemodynamic treatments on intra-operative and postoperative outcomes.

## Supplementary Material

Supplemental Digital Content
