# Supplementary material for: Blood pressure thresholds for the administration of balanced crystalloids and the effect on intra-operative hypotension: A proof-of-concept causal inference analysis featuring idealised dynamic treatment regimens
Source: Eur J Anaesthesiol. 2026 Feb 2;43(8):680–91. doi: 10.1097/EJA.0000000000002356 (PMC13336679; doi:10.1097/EJA.0000000000002356)
Supplement: Supplemental Digital Content [file ejanet-43-680-s001.docx]

**Supplementary Material**

**Blood pressure thresholds for the administration of balanced crystalloids and the effect on intraoperative hypotension: a proof-of-concept causal inference analysis featuring idealized dynamic treatment regimes**

Markus Huber*^1^, Hyung-Chul Lee^2^, Patrick Y. Wuethrich^1^

^1^ Department of Anaesthesiology and Pain Medicine, Inselspital, Bern University Hospital, University of Bern, Freiburgstrasse, 3010, Bern, Switzerland

^2^ Department of Anesthesiology and Pain Medicine, Seoul National University College of Medicine, Seoul National University Hospital, Seoul, South Korea

*Corresponding author:

Markus Huber, Dr. sc. ETH, Department of Anaesthesiology and Pain Medicine, Inselspital, Bern University Hospital, University of Bern, Bern, Switzerland, Freiburgstrasse 10, 3010 Bern, Switzerland, Tel: +41 31 664 12 15, Email: [markus.huber@insel.ch](mailto:markus.huber@insel.ch)

**Supplementary Figures**


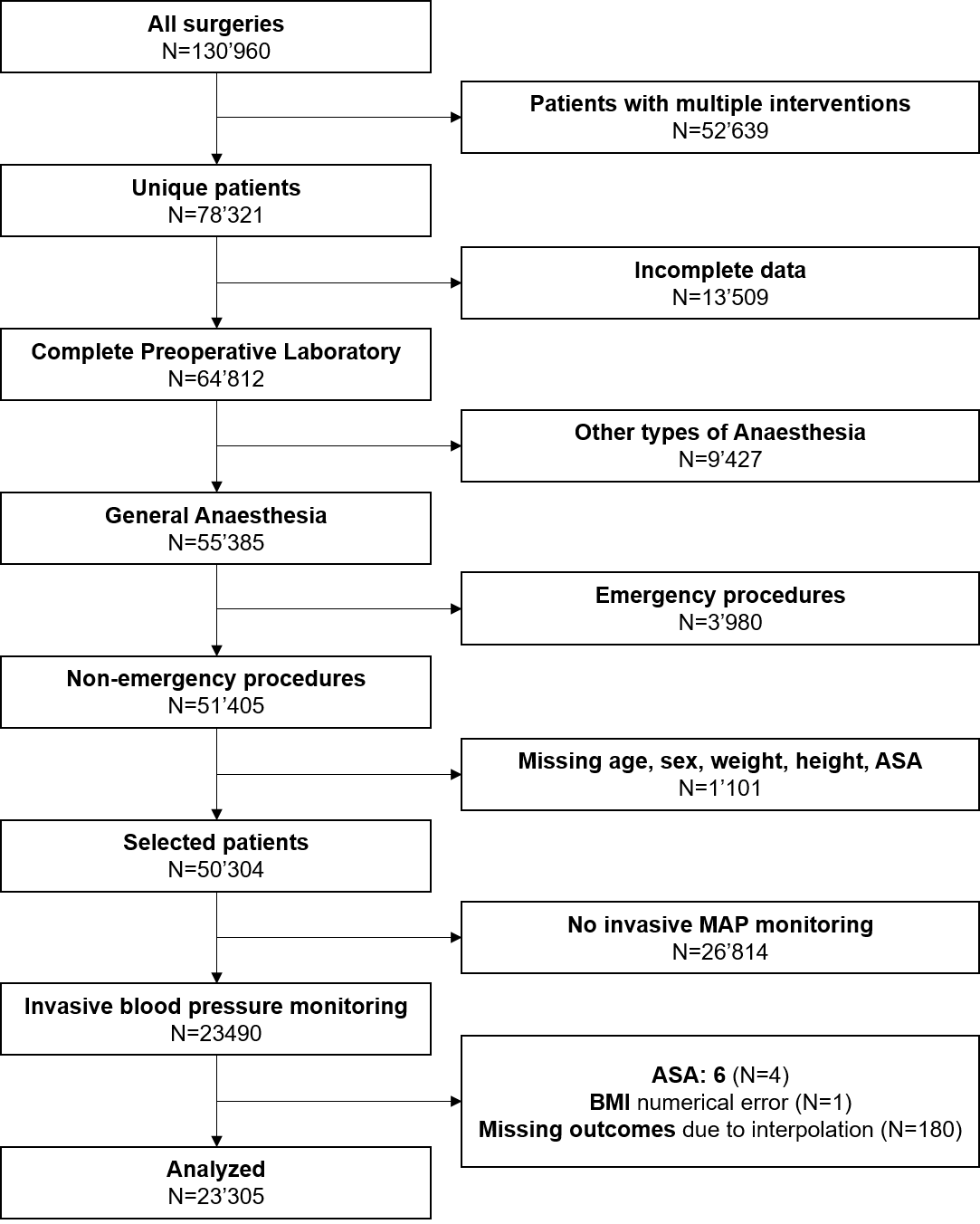


**Supplementary Figure SM1**. Flowchart illustrating data selection from the entire dataset to the final data selected for analysis.

**Computation of Dynamic Treatment Regimes**


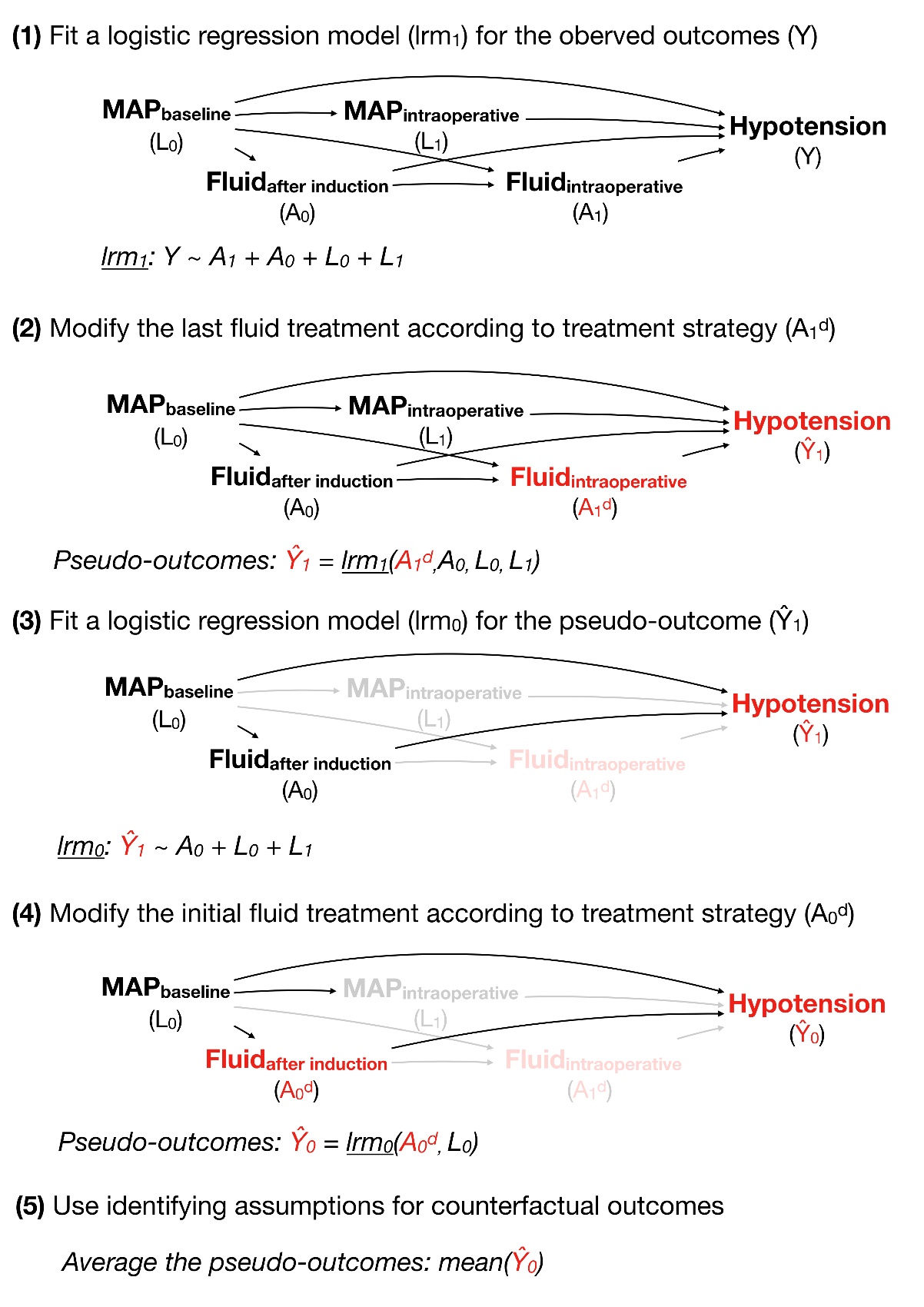


**Supplementary Figure SM2**. A simplified scenario of hemodynamic management to illustrate the effect of a dynamic treatment regime in fluid administration on hypotension. The illustration is based on the G-computation algorithm and is a simplified version of an example by Hoffmann et al. ^1^

Supplementary Figure SM4 explains the steps involved in assessing the impact of a DTR with fluid administration (a binary treatment for simplicity, denoted as A) at two time points (after induction and once intraoperatively) based on MAP values (the time-varying confounder L) at baseline and once intraoperatively on the postoperative outcome hypotension (Y). The illustration is based on the so-called G-computation algorithm and is a simplified version of an example by *Hoffmann et al*.:^1^

- First, a logistic regression model (*lrm_1_*) is fit with all available treatments and covariates, computing the conditional expectation of hypotension given the observed treatments and MAP measurements.
- Second, the observed fluid administration (*A_1_*) is changed according to the dynamic treatment regime (*A_1_^d^*) – for instance, giving no fluids if the previous MAP measurement was sufficiently high. Evaluating the fitted regression model *lrm_1_* with these modified treatment values results in new, counterfactual values for hypotension, so-called pseudo-outcomes ($\hat{Y}$_1_). The $\hat{Y}$_1_ thus represent the outcomes that would have been observed if the clinician had treated the patient intraoperatively according to the (counterfactual) treatment defined in the DTR.
- Third, another logistic regression model (*lrm_0_*) with the counterfactual values as outcomes is fit using the initial treatment and MAP values measured at baseline and intraoperatively.
- Fourth, evaluating the model *lrm_0_* with the fluid treatment after induction specified by the DTR (*A_0_^d^)* results in updated, counterfactual values for hypotension ($\hat{Y}$_0_).
- Fifth, the incidence of hypotension under the counterfactual treatments defined by the DTR can be derived by averaging the pseudo-outcomes $\hat{Y}$_0_. Note that the values of the time-varying confounders are not modified over the course of these computations.


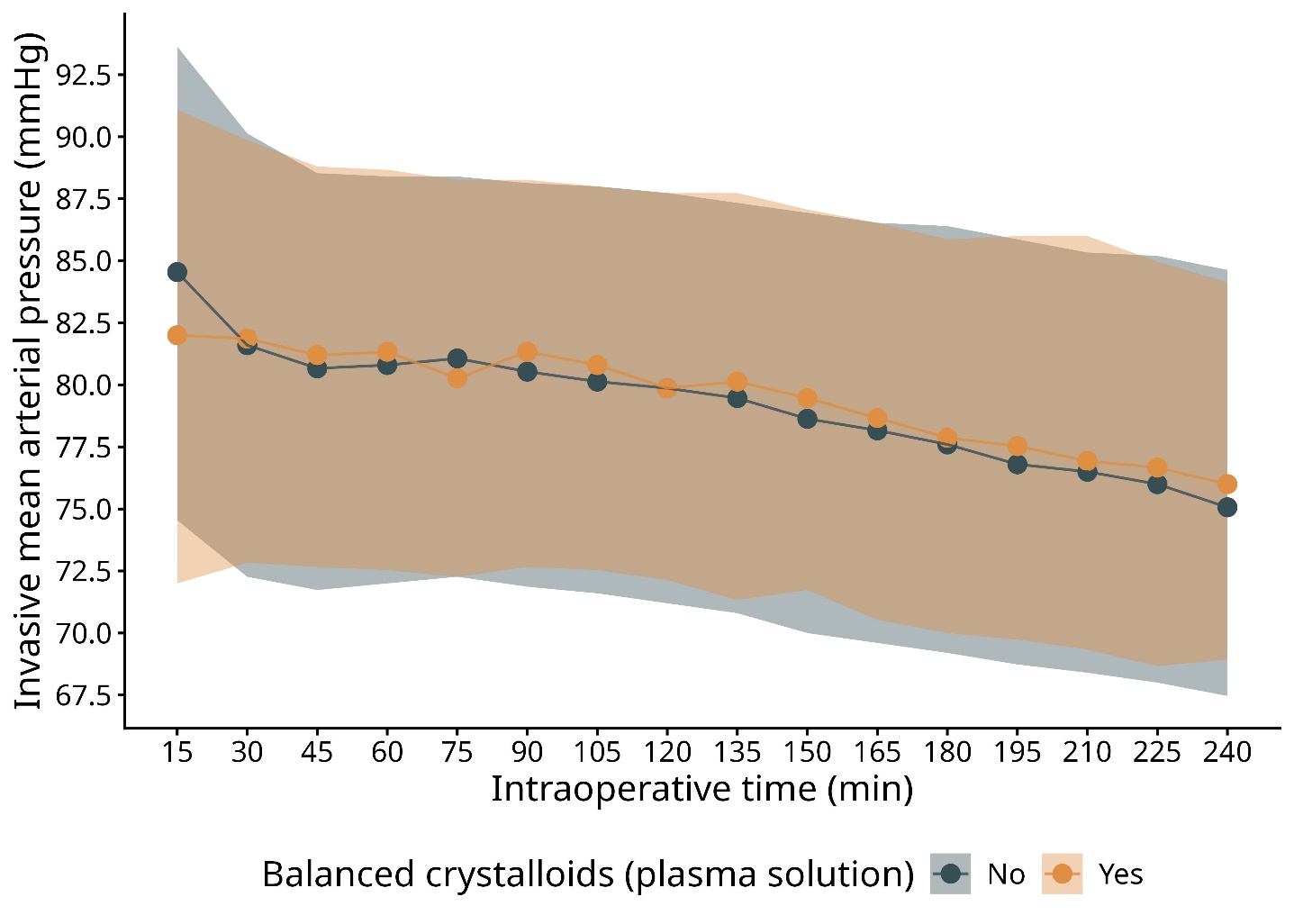


**Supplementary Figure SM3**. Summary measures of intraoperative invasive mean arterial pressure (median and interquartile ranges) stratified at each time point according to the administration of balanced crystalloids (plasma solution).

|  | **All** | **General surgery** | **Cardiothoracic surgery** | **Neurosurgery** | **Obstetrics and gynaecology** | **Urology** | **Orthopaedic surgery** | **Other*^†^*** |
| --- | --- | --- | --- | --- | --- | --- | --- | --- |
|  | ***N=23305 (100%)*** | ***N=6293 (27%)*** | ***N=4603 (20%)*** | ***N=4530 (19%)*** | ***N=3350 (14%)*** | ***N=2135 (9%)*** | ***N=1433 (6%)*** | ***N=961 (4%)*** |
| **Age** (years) | 60.0 [45.0;70.0] | 60.0 [50.0;70.0] | 60.0 [55.0;70.0] | 55.0 [45.0;65.0] | 45.0 [40.0;55.0] | 65.0 [55.0;70.0] | 65.0 [55.0;70.0] | 55.0 [45.0;65.0] |
| **Sex** (female) | 12233 (52.5%) | 2711 (43.1%) | 1950 (42.4%) | 2477 (54.7%) | 3350 (100.0%) | 415 (19.4%) | 783 (54.6%) | 547 (56.9%) |
| **ASA status:** |  |  |  |  |  |  |  |  |
| 1 | 6745 (28.9%) | 1674 (26.6%) | 786 (17.1%) | 1435 (31.7%) | 1582 (47.2%) | 582 (27.3%) | 393 (27.4%) | 293 (30.5%) |
| 2 | 13541 (58.1%) | 3779 (60.1%) | 2564 (55.7%) | 2777 (61.3%) | 1645 (49.1%) | 1387 (65.0%) | 873 (60.9%) | 516 (53.7%) |
| 3 | 2881 (12.4%) | 816 (13.0%) | 1188 (25.8%) | 301 (6.6%) | 117 (3.5%) | 164 (7.7%) | 157 (11.0%) | 138 (14.4%) |
| 4 | 133 (0.6%) | 20 (0.3%) | 64 (1.4%) | 17 (0.4%) | 6 (0.2%) | 2 (0.1%) | 10 (0.7%) | 14 (1.5%) |
| 5 | 5 (<0.1%) | 4 (0.1%) | 1 (<0.1%) | 0 (0.0%) | 0 (0.0%) | 0 (0.0%) | 0 (0.0%) | 0 (0.0%) |
| **Body mass index** (kg/m^2^) | 23.4 [21.5;26.0] | 22.9 [20.8;25.7] | 23.4 [21.2;25.7] | 24.2 [22.0;26.7] | 22.9 [20.8;25.7] | 24.2 [22.2;26.1] | 24.2 [22.0;26.7] | 23.9 [21.4;26.1] |
| **Surgery duration** (min) | 160.0 [105.0;250.0] | 200.0 [135.0;265.0] | 175.0 [110.0;315.0] | 160.0 [100.0;245.0] | 115.0 [80.0;168.8] | 130.0 [100.0;185.0] | 150.0 [95.0;210.0] | 190.0 [80.0;415.0] |
| **Anesthesia duration** (min) | 210.0 [145.0;300.0] | 240.0 [175.0;315.0] | 235.0 [155.0;380.0] | 220.0 [150.0;305.0] | 155.0 [115.0;210.0] | 170.0 [135.0;230.0] | 200.0 [145.0;255.0] | 230.0 [110.0;460.0] |
| **Hospitalisation** (days) | 8.0 [6.0;12.0] | 11.0 [9.0;15.0] | 9.0 [6.0;15.0] | 7.0 [5.0;9.0] | 6.0 [5.0;8.0] | 6.0 [6.0;7.0] | 9.0 [8.0;13.0] | 8.0 [4.0;11.0] |
| **Arterial mean blood pressure** (mmHg) | 80.9 [74.2;87.3] | 82.1 [76.3;87.9] | 77.3 [69.7;85.0] | 77.8 [71.8;84.3] | 85.5 [79.7;91.8] | 84.6 [79.4;90.1] | 80.3 [75.2;85.7] | 74.4 [68.7;80.0] |
| **Heart rate** (/min) | 66.8 [59.3;75.0] | 71.1 [63.1;79.3] | 68.2 [61.8;75.4] | 62.8 [55.6;71.1] | 63.5 [56.9;71.3] | 64.0 [57.8;71.4] | 67.6 [60.1;74.9] | 67.7 [59.0;77.6] |
| **Cristalloids and colloids** (mL) | 900.0 [450.0;1500.0] | 1100.0 [650.0;1650.0] | 550.0 [250.0;1000.0] | 950.0 [450.0;1500.0] | 850.0 [500.0;1400.0] | 1000.0 [600.0;1500.0] | 1300.0 [700.0;1900.0] | 800.0 [200.0;1500.0] |

*^†^*Internal medicine, Ophthalmology, Paediatrics, Plastic surgery, Radiation oncology, Otolaryngology

**Supplementary Table SM3**. Demographics and selected surgery characteristics stratified according to department.

|  | **0-15 min** | **15-30 min** | **30-45 min** | **45-60 min** | **60-75 min** | **75-90 min** | **90-105 min** | **105-120 min** | **120-135 min** | **135-150 min** | **150-165 min** | **165-180 min** | **180-195 min** | **195-210 min** | **210-225 min** | **225-240 min** |
| --- | --- | --- | --- | --- | --- | --- | --- | --- | --- | --- | --- | --- | --- | --- | --- | --- |
|  | ***N=23305*** | ***N=23310*** | ***N=23051*** | ***N=22372*** | ***N=21332*** | ***N=20081*** | ***N=18542*** | ***N=16895*** | ***N=15291*** | ***N=13775*** | ***N=12370*** | ***N=11026*** | ***N=9831*** | ***N=8770*** | ***N=7768*** | ***N=6869*** |
| **Balanced Crystalloids** (Yes) | 1825 (7.8%) | 3559 (15.3%) | 3956 (17.2%) | 3730 (16.7%) | 3593 (16.8%) | 3723 (18.5%) | 3335 (18.0%) | 3032 (17.9%) | 2710 (17.7%) | 2611 (19.0%) | 2176 (17.6%) | 2014 (18.3%) | 1798 (18.3%) | 1584 (18.1%) | 1340 (17.3%) | 1186 (17.3%) |
| **Epinephrine** (Yes) | 3 (<0.1%) | 4 (<0.1%) | 4 (<0.1%) | 4 (<0.1%) | 9 (<0.1%) | 7 (<0.1%) | 13 (0.1%) | 19 (0.1%) | 18 (0.1%) | 21 (0.2%) | 21 (0.2%) | 22 (0.2%) | 20 (0.2%) | 21 (0.2%) | 26 (0.3%) | 21 (0.3%) |
| **Phenylephrin**  (Yes) | 938 (4.0%) | 1010 (4.3%) | 867 (3.8%) | 704 (3.1%) | 625 (2.9%) | 571 (2.8%) | 586 (3.2%) | 612 (3.6%) | 594 (3.9%) | 564 (4.1%) | 556 (4.5%) | 537 (4.9%) | 519 (5.3%) | 481 (5.5%) | 397 (5.1%) | 366 (5.3%) |
| **Ephedrine** (Yes) | 16 (0.1%) | 11 (<0.1%) | 9 (<0.1%) | 7 (<0.1%) | 2 (<0.1%) | 4 (<0.1%) | 11 (0.1%) | 16 (0.1%) | 9 (0.1%) | 16 (0.1%) | 18 (0.1%) | 19 (0.2%) | 27 (0.3%) | 22 (0.3%) | 14 (0.2%) | 17 (0.2%) |
| **Vasopressin** (Yes) | 1702 (7.3%) | 1683 (7.2%) | 1168 (5.1%) | 1006 (4.5%) | 868 (4.1%) | 848 (4.2%) | 779 (4.2%) | 729 (4.3%) | 608 (4.0%) | 576 (4.2%) | 533 (4.3%) | 429 (3.9%) | 393 (4.0%) | 335 (3.8%) | 294 (3.8%) | 239 (3.5%) |
| **Half normal saline** (Yes) | 4 (<0.1%) | 12 (0.1%) | 11 (<0.1%) | 12 (0.1%) | 12 (0.1%) | 17 (0.1%) | 14 (0.1%) | 18 (0.1%) | 10 (0.1%) | 21 (0.2%) | 19 (0.2%) | 8 (0.1%) | 12 (0.1%) | 16 (0.2%) | 22 (0.3%) | 17 (0.2%) |
| **Hartmann's solution** (Yes) | 1455 (6.2%) | 2733 (11.7%) | 3002 (13.0%) | 2634 (11.8%) | 2410 (11.3%) | 2647 (13.2%) | 2124 (11.5%) | 1834 (10.9%) | 1606 (10.5%) | 1632 (11.8%) | 1331 (10.8%) | 1110 (10.1%) | 974 (9.9%) | 940 (10.7%) | 742 (9.6%) | 626 (9.1%) |
| **Normal saline** (Yes) | 963 (4.1%) | 1222 (5.2%) | 1360 (5.9%) | 1370 (6.1%) | 1429 (6.7%) | 1294 (6.4%) | 1150 (6.2%) | 1111 (6.6%) | 1102 (7.2%) | 913 (6.6%) | 872 (7.0%) | 832 (7.5%) | 763 (7.8%) | 644 (7.3%) | 623 (8.0%) | 546 (7.9%) |
| **Hydroxyethyl-starch solution** (Yes) | 130 (0.6%) | 198 (0.8%) | 317 (1.4%) | 429 (1.9%) | 514 (2.4%) | 539 (2.7%) | 511 (2.8%) | 500 (3.0%) | 449 (2.9%) | 427 (3.1%) | 384 (3.1%) | 377 (3.4%) | 315 (3.2%) | 280 (3.2%) | 277 (3.6%) | 248 (3.6%) |
| **Transfused red blood cell** (Yes) | 4 (<0.1%) | 13 (0.1%) | 25 (0.1%) | 37 (0.2%) | 43 (0.2%) | 45 (0.2%) | 59 (0.3%) | 64 (0.4%) | 71 (0.5%) | 67 (0.5%) | 70 (0.6%) | 73 (0.7%) | 64 (0.7%) | 73 (0.8%) | 57 (0.7%) | 57 (0.8%) |
| **Transfused fresh frozen plasma** (Yes) | 1 (<0.1%) | 2 (<0.1%) | 6 (<0.1%) | 7 (<0.1%) | 16 (0.1%) | 12 (0.1%) | 14 (0.1%) | 24 (0.1%) | 27 (0.2%) | 23 (0.2%) | 24 (0.2%) | 26 (0.2%) | 44 (0.4%) | 39 (0.4%) | 37 (0.5%) | 38 (0.6%) |
| **Urine output** (>0 mL) | 1683 (7.2%) | 3182 (13.7%) | 4101 (17.8%) | 4494 (20.1%) | 4019 (18.8%) | 4303 (21.4%) | 3840 (20.7%) | 3757 (22.2%) | 3224 (21.1%) | 3264 (23.7%) | 2714 (21.9%) | 2681 (24.3%) | 2290 (23.3%) | 2258 (25.7%) | 1883 (24.2%) | 1780 (25.9%) |
| **Estimated blood loss** (>0 mL) | 1422 (6.1%) | 3903 (16.7%) | 5349 (23.2%) | 5847 (26.1%) | 5420 (25.4%) | 5128 (25.5%) | 4411 (23.8%) | 3946 (23.4%) | 3492 (22.8%) | 3013 (21.9%) | 2535 (20.5%) | 2292 (20.8%) | 1931 (19.6%) | 1679 (19.1%) | 1363 (17.5%) | 1234 (18.0%) |
| **Heart rate** (bpm) | 66.4 (12.1) | 66.8 (12.4) | 66.8 (12.5) | 66.5 (12.5) | 66.6 (12.6) | 67.2 (12.7) | 67.9 (12.8) | 68.7 (13.0) | 69.5 (13.1) | 70.3 (13.2) | 70.9 (13.2) | 71.6 (13.4) | 72.2 (13.4) | 72.9 (13.8) | 73.6 (13.8) | 74.2 (13.9) |
| **MAP** (mmHg) | 84.1 (13.0) | 81.6 (12.2) | 80.7 (11.8) | 80.6 (12.0) | 80.6 (12.0) | 80.5 (12.0) | 80.2 (11.9) | 79.9 (11.7) | 79.6 (11.9) | 79.0 (12.0) | 78.5 (12.0) | 78.1 (12.0) | 77.7 (12.2) | 77.5 (12.1) | 77.0 (12.2) | 76.4 (12.2) |
| **Hypotension** (<65 mmHg) | 4824 (20.7%) | 5490 (23.6%) | 5005 (21.7%) | 4476 (20.0%) | 4168 (19.5%) | 4009 (20.0%) | 3815 (20.6%) | 3658 (21.7%) | 3484 (22.8%) | 3420 (24.8%) | 3251 (26.3%) | 3081 (27.9%) | 2908 (29.6%) | 2664 (30.4%) | 2423 (31.2%) | 2292 (33.4%) |

**Supplementary Table SM4**. Overview of hemodynamic managements and hemodynamic quantities over the intraoperative course over 4 hours. For illustration purposes only and to ease interpretation, most quantities were dichotomized. For example, urine output was dichotomized into patients whose output was non-zero and the administration of Hartmann's solution was dichotomized into the categories administered (“Yes”) and not administered (“No”). Note that the model used to compute the dynamic treatment regimes features the actual, numerical values.

**
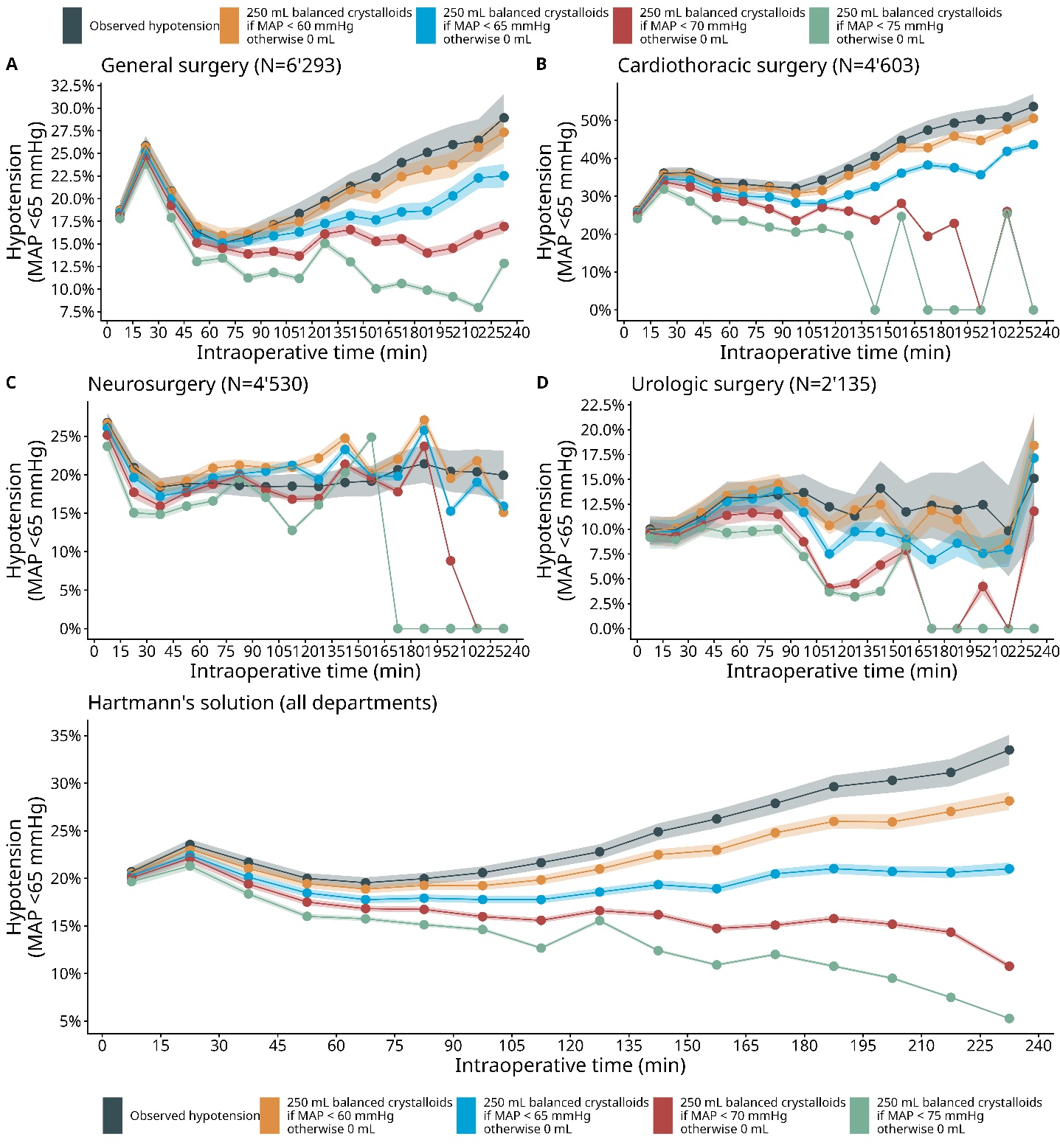
**

**Supplementary Figure SM5**. Sensitivity analysis of Figure 5 of the main manuscript, but for different surgical departements (panels A-D) as well as for Hartmann’s solution as the intervention instead of plasma solution (E; including all departments).

**References**

1. Hoffman KL, Salazar-Barreto D, Williams NT, Rudolph KE, Díaz I. Studying Continuous, Time-varying, and/or Complex Exposures Using Longitudinal Modified Treatment Policies. *Epidemiology*. 2024;35(5):667-675. doi:10.1097/ede.0000000000001764
